# A simple data-adaptive probabilistic variant calling model

**DOI:** 10.1186/s13015-015-0037-5

**Published:** 2015-03-04

**Authors:** Steve Hoffmann, Peter F Stadler, Korbinian Strimmer

**Affiliations:** Junior Research Group Transcriptome Bioinformatics, University Leipzig, Härtelstraße 16-18, Leipzig, Germany; Interdisciplinary Center for Bioinformatics and Bioinformatics Group, University Leipzig, Härtelstraße 16-18, Leipzig, Germany; LIFE Research Center for Civilization Diseases, University Leipzig, Härtelstraße 16-18, Leipzig, Germany; Department of Theoretical Chemistry, University Vienna, Währinger Straße 17, Vienna, Austria; RNomics Group, Fraunhofer Institute for Cell Therapy and Immunology – IZI, Perlickstraße 1, Leipzig, Germany; Max-Planck-Institute for Mathematics in the Sciences, Inselstraße 22, Leipzig, Germany; Center for non-coding RNA in Technology and Health, University of Copenhagen, Grønnegårdsvej 3, Frederiksberg, Denmark; Santa Fe Institute, 1399 Hyde Park Road, Santa Fe, USA; Institute for Medical Informatics, Statistics and Epidemiology, University of Leipzig, Härtelstraße 16–18, Leipzig, D-04107 Germany; Department of Epidemiology and Biostatistics, Imperial College London, Norfolk Place, London W2 1PG, UK

## Abstract

**Background:**

Several sources of noise obfuscate the identification of single nucleotide variation (SNV) in next generation sequencing data. For instance, errors may be introduced during library construction and sequencing steps. In addition, the reference genome and the algorithms used for the alignment of the reads are further critical factors determining the efficacy of variant calling methods. It is crucial to account for these factors in individual sequencing experiments.

**Results:**

We introduce a simple data-adaptive model for variant calling. This model automatically adjusts to specific factors such as alignment errors. To achieve this, several characteristics are sampled from sites with low mismatch rates, and these are used to estimate empirical log-likelihoods. The likelihoods are then combined to a score that typically gives rise to a mixture distribution. From this we determine a decision threshold to separate potentially variant sites from the noisy background.

**Conclusions:**

In simulations we show that our simple model is competitive with frequently used much more complex SNV calling algorithms in terms of sensitivity and specificity. It performs specifically well in cases with low allele frequencies. The application to next-generation sequencing data reveals stark differences of the score distributions indicating a strong influence of data specific sources of noise. The proposed model is specifically designed to adjust to these differences.

## Background

Recent studies report a strikingly low concordance of currently available methods and pipelines for identification of single nucleotide variation (SNV), both somatic and germline, indicating that computational methods as well as sequencing protocols have a major impact on the sensitivity and specificity of the variation calling tool [1]. Specifically, the allelic fraction as well as the coverage of the variant allele are crucial determinants for the statistical benchmarks [2,3]. Practical guidelines of SNV callers such as GATK [4] or SAMtools [5] suggest to apply rigorous postprocessing filters to reduce the number of false positive calls. Other studies indicate that the application of these filters lead to a substantial improvement of the concordance of the callers [6]. Nevertheless, applying stringent thresholds for variables such as the strand bias, the coverage or read start variation bears the risk of losing important information [7]. These authors emphasize that the different algorithmic and statistical components of a variant caller have to be evaluated as a whole and cannot not be meaningfully judged as single components.

**Figure 1:**
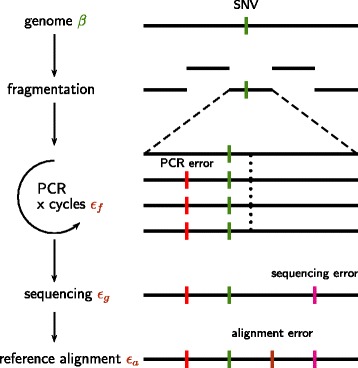
**Accumulation and sources of errors in next generation sequencing reads.** The identification of single nucleotide variations (green dashes) is complicated by various sources of error. PCR errors accumulate during the amplification and sequencing step. After fragmentation single fragments undergo several amplification cycles. Errors are introduced with a rate *δ*_*f*_ (red dashes). Further errors are accumulated during sequencing (*δ*_*g*_). During the alignment to the reference further mismatches and indels are introduced (*δ*_*a*_).

If DNA library preparation protocols and sequencing machines were able to produce error-free and unbiased sequences of sufficient length the task of variant calling would be easy. Due to various error sources and technical limitations of library preparation, sequencing, and alignment, however, a substantial level of noise complicates the analysis. Since these factors can not be totally controlled during the experiment it seems reasonable to adjust the thresholds for calling a variant depending on the separability of noise and signal, i.e. the true variants. During amplification incorrect nucleotides are incorporated with some error rate *ε*_*f*_ and during the sequencing step incorrect nucleotides are called with the rate *ε*_*g*_. After the alignment of the reads to a reference sequence we may observe these errors as mismatches or indels. Additional mismatches and indels are caused by these reference and alignment errors (*ε*_*a*_). The mismatch rate of a genomic site can be assumed to be the sum *δ*=*ε*+*β*, where *β* represents the biological variation and *ε* is the compound effect of the technical errors *ε*_*f*_, *ε*_*g*_, and *ε*_*a*_. Figure 1 summarizes this situation.

The two most commonly used tools for SNV calling methods, SAMtools and GATK, employ probabilistic models for variant calling. Specifically, the algorithm used by SAMtools [8] is based on the likelihood of a genotype which is computed as

(1) $$ \begin{aligned} L(g) =& \frac{1}{m^{k}} \prod\limits^{l}_{j=1} \left[(m-g)\zeta_{j}+g(1-\zeta_{j})\right]\\ &\prod\limits^{k}_{j=l+1} \left[(m-g)(1-\zeta_{j})+g\zeta_{j}\right]\,, \end{aligned}  $$

where *g* denotes the number of reference alleles, *m* the ploidy, *k* the number of reads seen at a site, and *ζ* the error probability delivered by the sequencer. Eq. 1 assumes that the first *l* bases are identical to the reference, the subsequent bases are not. Subsequently, from this a likelihood for the allele count *L*(*c*) is obtained. Using the observed allele frequency spectrum *ϕ*_*c*_ as prior information a posterior probability

(2) $$ \Pr\{ c \} = \frac{\phi_{c} L(c)} {\sum_{c^{\prime}} \phi_{c^{\prime}} L({c^{\prime}})}  $$

is computed, and a variant is called if Pr{*c*>1} exceeds a certain specified threshold.

The GATK pipeline uses a related probabilistic model for calling variants [9]. Similar to SAMtools, the probability Pr{*D*_*j*_|*A*} of observing the base *D*_*j*_ under the hypothesis that *A* is the true base is calculated by

(3) $$  \Pr\{D_{j} | A\} =\left\{ \begin{aligned} &1 - \zeta_{j} & D_{j} = A\\ &\zeta_{j} \; \Pr\{\text{A is true} | \text{\(D_{j}\) is miscalled}\} & \text{otherwise,} \end{aligned} \right.  $$

where Pr{A is true|*D*_*j*_ is miscalled} is a precomputed, sequencer specific lookup table. Using prior information based on precomputed heterozygosity estimations GATK evaluates the posterior probabilities of a site to be variant. As with SAMtools calls are determined using fixed preselected thresholds.

Here, we propose a simple probabilistic model for variant calling using a data adaptive threshold on the scale of log-odd-ratios computed from empirical distributions of certain site characteristics. Our approach allows to optimally separate simulated SNVs from the noisy background without specification of a threshold for posterior probabilities. In brief, our model starts out by evaluating the mismatch frequencies *δ* in a data set. Subsequently, we sample several characteristics of the sites with small *δ* to serve as empirical reference model. The fundamental idea used here is that the vast majority of sites is invariant and thus allows to capture the features of the data specific error model. These characteristics are then used to form empirical log-likelihoods that are combined to a log-odds type score. Typically, we observe a mixture distribution of two score populations, which we may then separate by a decision threshold.

Next, we discuss the details of our approach and the proposed data-adaptive variant calling algorithm. Subsequently, we apply our method to both synthetic and next generation sequencing data from various species. A reference implementation in C99 of our method called haarz is available at http://www.bioinf.uni-leipzig.de/software.html.

## Methods

### Notation

We denote the position of a site in the reference genome by *i*∈[1,…,*n*] where *n* is the genome length. After aligning the reads to a genome each reference base typically has several nucleotides aligned to it. We refer to the set of all aligned nucleotides as the *cross sectionC*^*i*^ at position *i*. The coverage at position *i* is the size |*C*^*i*^| of this set. We use the index *j*∈[1,…,|*C*^*i*^|] to refer to a specific read. The length of read *j* aligned to site *i* is denoted by ${m^{i}_{j}}$, and the position of a nucleotide in a read is denoted by ${k^{i}_{j}} \left \{1, \ldots, {m^{i}_{j}}\right \}$. For simplicity, we occasionally leave out the index *i* when there is no danger of ambiguity.

The nucleotide in a cross section can be partitioned into sets of match (*M*) and mismatch ($\overline {M}$) nucleotides so that $C^{i} = M^{i} \cup \overline {M}^{i}$. The variant calling algorithm described below uses the partition $\{ M^{i}, \overline {M}^{i} \}$ at each position *i* to compute an overall score for this particular site.

### Biological versus technical variation

The *mismatch rateδ*^*i*^=|*M*^*i*^|/|*C*^*i*^| is the observed number of mismatches divided by the coverage. The mismatch rate *δ*^*i*^=*β*^*i*^+*ε*^*i*^ may be decomposed into biological and technical variation, where *ε*^*i*^ denotes the technical error that accumulated during the preparation, sequencing and alignment steps and *β*^*i*^ denotes the biological nucleotide variation at site *i*.

We aim to distinguish biologically variant positions (*β*^*i*^>0) from non-variant positions (*β*^*i*^=0), based on the observed mismatch rates *δ*^*i*^ and site characteristic scores derived from sequence data or produced during sequencing.

We assume that cross sections with high mismatch rates are indicative of biological variation in the sample, whereas in cross sections with small mismatch rate the mismatches are more likely due to technical errors. Conversely, in the overwhelming majority of cross sections *C*^*i*^ we may assume that there is no biological variation present, i.e. *β*^*i*^=0, and thus mismatches are only caused by technical errors.

For use in the variant calling score we estimate for each *δ*^*i*^ the corresponding empirical quantile *q*(*δ*^*i*^). The motivation for using the quantile rather than the actual value is that it implements a simple normalization of the error. The empirical quantile *q*(*δ*^*i*^) is estimated by tabulating the cumulative frequencies of *δ*^*i*^ across the genome and then reading off the quantile from the resulting empirical distribution function (ECDF).

To ascertain the probabilities of certain site characteristics, discussed further below, we uniformly sample from sites with 0<*δ*^*i*^<0.5. Informally, these characteristics then reflect “background distributions” of non-variant sites and thus are estimated from sites with less than 50% of mismatches.

The degree of biological variation depends on the type of genome. For heterozygous genomes one expects to find predominantly SNP alleles with *β*^*i*^=0.5 or *β*^*i*^=1.0, whereas cancer tissues may show mutations with 0<*β*^*i*^≤1 depending on the heterogeneity and cancer cell content of the sample. Similarly, arbitrary values of *β*_*i*_ will appear in whole population sequencing data. Accordingly, we expect different values of *β*^*i*^ for mixtures of sequencing data from different individuals. The variant calling algorithm introduced in the following makes no assumptions concerning the presence of diploid genomes, knowledge about the ploidy, homo- or heterozygosity.

### Site characteristics

In addition to the partitioning of nucleotides at a given site into match and mismatch sets, our algorithm uses the following information, which is typically reported by the sequencer or the read mapper for every site *i* and read *j*:

- the nucleotide qualities (*Q*),
- relative read position (*P*),
- errors in the alignment (*R*), and
- the number of multiple hits (*H*).

The nucleotide qualities take on values between 0 and 1 and are given as *Q*=1−*ζ*, i.e., as probability of a base being correct, with values close to 1 corresponding to optimally accurate sequencing. We directly use *Q* in computing the variant calling score.

The relative read position is given by $P = {k^{i}_{j}} /{m^{i}_{j}}$. For the construction of our variant calling score we employ the probability Pr(*M*|*P*) of a match at a given read position, along with the maximum *P*_*M*_= max*P* Pr(*M*|*P*). The probability of a mismatch is then given by $\Pr (\overline {M}|P) = 1 - \Pr (M|P)$, and its maximum $P_{\overline {M}}= \max _{P} \Pr (\overline {M}|P)$. We estimate the probability Pr(*M*|*P*) empirically, i.e., by appropriately counting matches and mismatches over all sites and reads.

The number of errors in the alignment is an integer value greater or equal to zero, and denoted here by *R*. Finally, the number of multiple hits *H* describes the number of alignments for each read. The multiplicity of an alignment yields information on the repetitiveness of a genomic region. As above for the relative read position, we tabulate the occurrence of matches for each value of *R* and and *H* and correspondingly obtain estimates of the probabilities Pr(*M*|*R*) and Pr(*M*|*H*).

### Scores for distinguishing variant and non-variant sites

Informally, in a non-variant cross section (*β*^*i*^=0) we expect that the probability of a match base increases with high nucleotide qualities (good sequencing), low read error rates, few multiple hits and good read positions. Conversely, the probability of mismatching bases in non-variant cross sections increases with low nucleotide qualities (poor sequencing), high read error rates, multiple hits and error-prone read positions. For variant sites with *β*>0 we expect to have high nucleotide qualities, good read positions and few multiple hits also for the mismatch bases. Consequently, for distinguishing variant from non-variant sites only the mismatching bases are relevant.

We introduce four log-odds ratios to formalize and summarize the evidence for a variant over a non-variant based on the above four site characteristics.

$$\Delta_{Q} = \frac{1}{|C|} \sum_{x\in\overline{M}} \log{Q_{x}\over 1-Q_{x}} $$

for base qualities,

$$\Delta_{P} = \frac{1}{|C|} \sum_{x\in\overline{M}} \left(\log{\Pr(M|P_{x}) \over 1-\Pr(M|P_{x})} + \log{P_{\overline{M}} \over P_{M}} \right) $$

for read positions, which are rescaled by their respective maximally attained values *P*_*M*_ and $P_{\overline {M}}$,

$$\Delta_{R} = \frac{1}{|C|} \sum_{x\in\overline{M}} \log{\Pr(M|R_{x}) \over 1-\Pr(M|R_{x})} $$

for read errors, and

$$\Delta_{H} = \frac{1}{|C|} \sum_{x\in\overline{M}} \log{\Pr(M|H_{x}) \over 1-\Pr(M|H_{x})} $$

for multiple matches. Note that only reads with mismatching bases in a cross-section are used for estimation, i.e. match bases are ignored. If there are only match bases in a cross-section, i.e. if $| \overline {M} |=0$ then the cross-section *is not considered in any component of our model*.

### Variant calling with adaptive threshold

From these log-odds ratios we now construct a total score for variant calling by computing, at any position *i*,

$$S_{i} = \Delta_{P_{i}} + \Delta_{Q_{i}} + \Delta_{R_{i}} + \Delta_{H_{i}} + \log q(\delta^{i})\,. $$

This score comprises the four summaries of the site characteristics, as well as the log-quantile of the observed mismatch rate *δ*^*i*^, i.e. the observed number of changes at position normalized by coverage. A low quantile for *δ*^*i*^ thus strongly penalizes the overall score.

For variant calling we now proceed as follows. We assume that the majority of the sites are non-variant, and only a smaller part is variant, with *S*_*i*_>0. Thus, the observed distribution of *S*_*i*_ will be a mixture distribution, consisting of a null distribution corresponding to the invariant sites and an alternative “contamination” component corresponding to the variant sites.

In order to find an optimal adaptive cut-off separating the background from potential variants we estimate the densities by fitting a natural spline using Poisson-regression to the histogram, following the procedure described by [10].

Subsequently, we numerically find the location with the minimum density and use it as threshold for separating the two score populations. Specifically, we fit natural splines to the histogram for *S*_*i*_>0 and numerically determine the local minimum. If there are multiple minima the leftmost minimum is used. The corresponding threshold is denoted by *S*^∗^.

In some cases there is no minimum in the histogram of the empirical scores. In this case we use as fall-back solution the upper 95% quantile as threshold. A missing minimum might indicate that the score model does not suffice to reliably call the variants.

**Figure 2:**
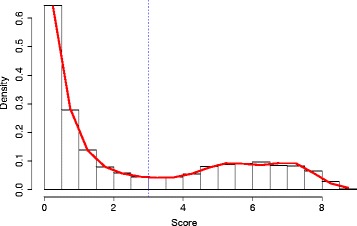
**Adaptive cutoff determination.** The figure shows the distribution of the empirical scores *S*_*i*_>0 (red continuous line) and the cutoff *S* ^∗^ (dotted blue vertical line). The threshold separates potential variants from background signal.

Once the threshold *S*^∗^ is established, we declare all sites *S*_*i*_>*S*^∗^ to be variant. In Figure 2 this procedure is illustrated using data from *A. thaliana*.

We note that by construction of the score *S*_*i*_ we assume independence of the site characteristics. However, in practice there will be correlation, and as alternative one may also consider a fully multivariate construction of the score *S*_*i*_. However, this is not without its own drawbacks, as the correlation among site characteristics may be hard to estimate reliably. Moreover, as is well known from classification and “naive Bayes” analysis, independence models are typically rather robust and often even outperform more complex parameter-rich multivariate models.

## Results and discussion

### Simulation study

To evaluate the reference implementation “haarz” of our adaptive model we compared it with the two frequently used SNV callers GATK [4] and SAMtools [5]. The precise command line settings are summarized in the Appendix.

**Figure 3:**
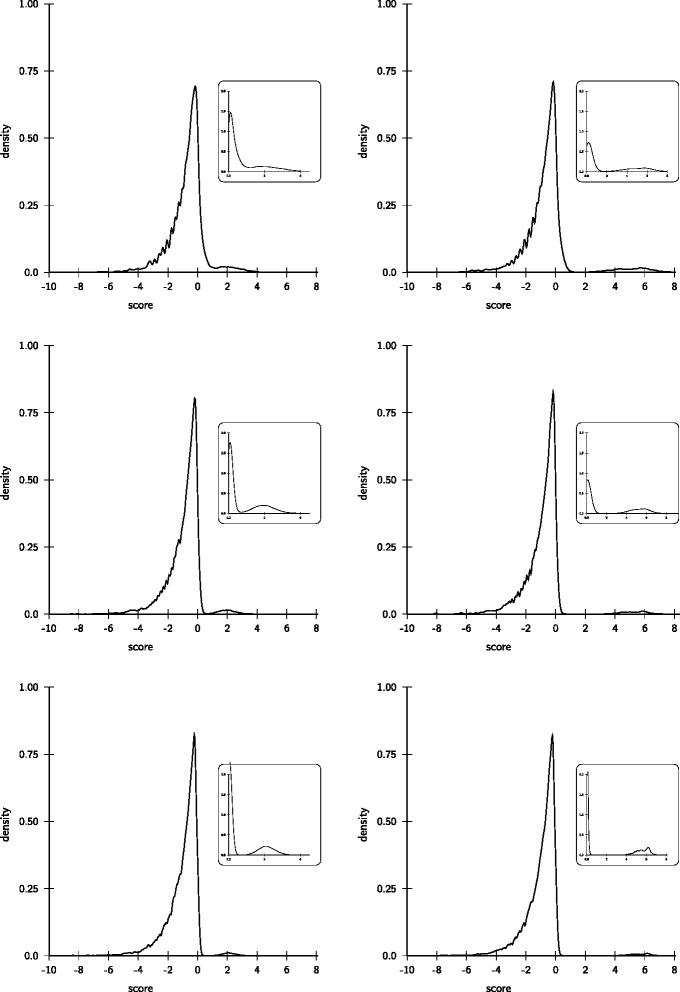
**Score distributions of simulated SNV data.** The left (right) column shows the score distributions of simulated SNVs with 20% (50%) variant allele content for 20-fold (top), 50-fold (middle) and 100-fold (bottom) coverage. The insets show the density of scores >0. With the increase of coverage a population of scores >0 is clearly distinguishable from the background.

We simulated next generation sequencing data for the human chromosome 21 using GemSIM (version 1.6) [11] with the default model and coverages ranging from 10, 20, 30, 50, 100, to 200-fold. The simulated content of the variant allele was either 0.2 or 0.5. Simulated read length was 100. For mapping we used the aligners that are recommended for each method. Specifically, we used BWA [12] to generate the alignments for GATK and SAMtools. For the reference implementation of our model we used segemehl [13]. After mapping and variant calling we collected for each combination of coverage and variant allele frequencies the number of false positives (FP), true positives (TP), false negatives (FN), and true negatives (TN). From this we computed the recall (sensitivity) *SENS*=*TP*/(*TP*+*FN*) and the positive predictive value *PPV*=*TP*/(*FP*+*TP*), i.e. the true discovery rate. For the proposed data adaptive model we investigated the score distribution for all 12 experiments (Figure 3). Except for the combination of low coverage (10 ×) and low variant allele content (20%) we observe the presence of two populations. The separability of these score populations improves with increasing coverage and variant allele content. In each case, the minimum score for variant calls was automatically set to the value where the density of scores >0 attains its first first local minimum. Subsequently all positions with a score equal or greater were called as SNV and compared to the other callers.

**Figure 4:**
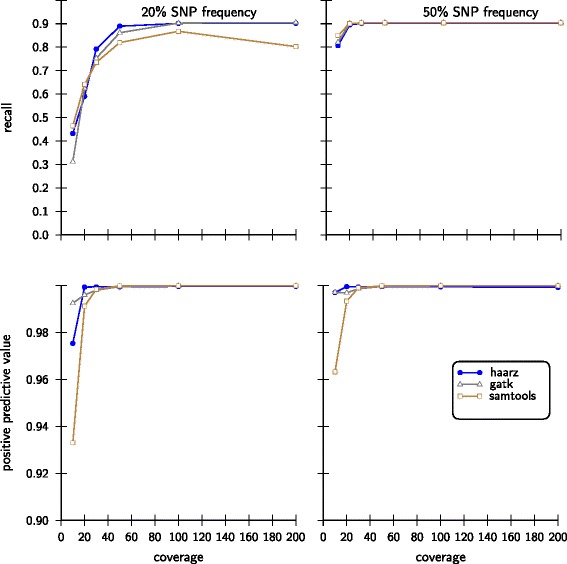
**Statistical performance measures on simulated data sets.** The data adaptive model implemented in haarz was compared to SAMtools and GATK in terms of recall and positive predictive value. SNV calling was performed on twelve different data sets varying in the content of the variant allele (20% and 50%) as well as the simulated coverage (10-200). In all of these scenarios the data adaptive model is at par with both alternative callers.

All of the tested programs show a good recall and positive predictive value in all 12 simulations. For low allele contents in conjunction with low coverages, however, SAMtools attains comparably low positive predictive values. Surprisingly, after reaching a maximum recall for the coverage of 100, the recall drops substantially for coverage 200. For the simulations with 50% allele content, all tools show high recalls and good positive predictive values. Again, SAMtools achieves only a comparably low positive predictive value for poorly covered SNVs (Figure 4). Except for the lowest coverage, all tools performed well on these data sets.

**Figure 5:**
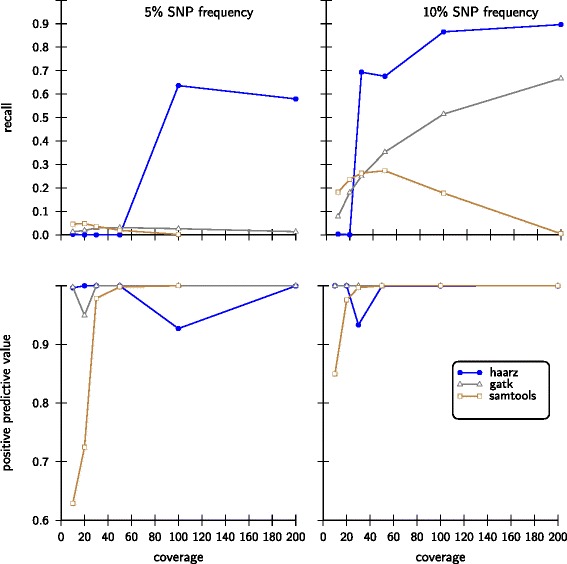
**Statistical performance measures on simulated data sets with small variant allele frequency.** As in Figure 4 we compare our approach with SAMtools and GATK with 10% and 5% of minor allele frequencies.

In Figure 5 we show results for the challenging case of small minor allele frequencies of 5% and 10%. Our approach compares well in these rather difficult cases, in contrast to SAMtools and GATK. For the low coverages, our algorithm does not find a clear cutoff and thus resorts to the 95% criteria. Since there are very few sites with high scores, i.e. *S*>0, the recall is low and the positive predictive value is high. As soon as higher coverages are reached and a minimum is found, the recall is increases substantially. We note that for low SNP frequencies in conjunction with low coverages the sample sizes for sampling site characteristics (default sample size: 100000; see Appendix) need to be increased to calculate the distribution of scores *S*>0.

### Application to data sets

The good overlap between the different methods in our simulation study as well as the small number of false positives is in stark contrast to the experience of greatly differing variant calls in real life data (e.g. [1]). We therefore applied our model to diverse real data from both diploid and haploid organisms.

Paired end next generation sequencing data for *Arabidopsis thaliana* (SRR519713), *Escherichia coli* (ERR163894) and *Drosophila melanogaster* (SRR1177123) was downloaded under the respective accession numbers from the Short Read Archive (www.ncbi.nlm.nih.gov/sra). The *Arabidopsis* data was aligned to the reference genome version 10.5. With the data set SRR519713 we obtained a coverage of ∼30-fold. The *E. coli* data set was aligned to the reference genome *E. coli k12* assembly v1.16. With ERR163894 we obtained a coverage of ∼60-fold. Finally, SRR1177123 was aligned to the *D. melanogaster* reference version dm3. The coverage was ∼25-fold. For the alignments, calling and filtering we used standard parameters. Precise settings are given in the Appendix.

**Figure 6:**
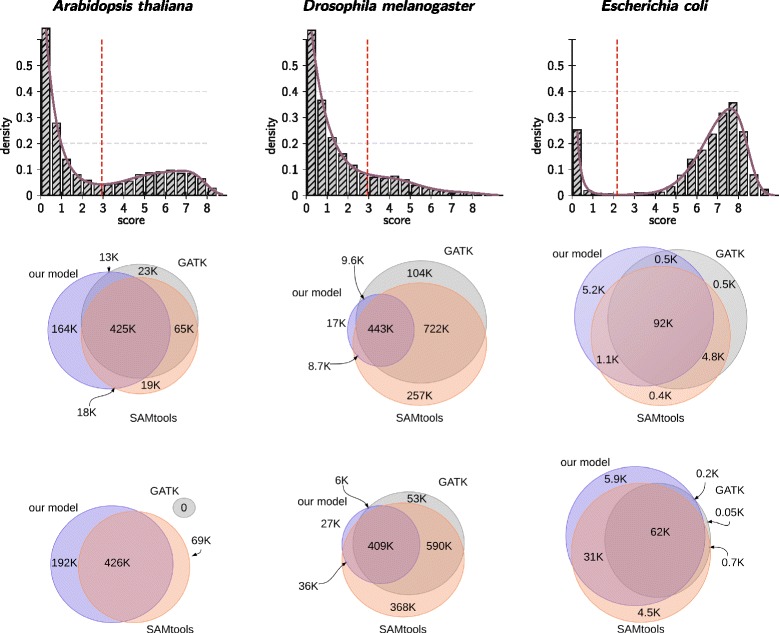
**Score distributions and congruence of variant calls in next generation sequencing data.** While a clear separation of score populations is observable for the diploid *A. thaliana* and the haploid *E. coli*, only a shallow minimum can be observed in case of *D. melanogaster*. Hence, in the latter case our model automatically adjusts to the data such that the calling appears to be more conservative: less than 4 percent of the calls are not supported by any caller and 92% of its calls are supported both by GATK and SAMtools. On the other hand, our model calls more variants in the other two cases.

The score distributions are shown in first line of Figure 6. In the case of the plant *A. thaliana* and the procaryote *E. coli*, a clear separation of two populations is observable. On the other hand, the separation of the score populations in *D. melanogaster* data set is less pronounced.

In the lower part of Figure 6 we show the concordance of variant calls of three investigated methods for the three data sets. For the well separable cases, the number of calls made by the data adaptive model is equal or higher as compared to the two competing callers. For the *D. melanogaster* data set our approach is more conservative and reports fewer variants. Most of these, however, are also found by SAMtools and GATK. About 92% percent of the calls from our model are also supported by both of the other callers and only 4% are not supported by any of the two alternative approaches. From the score distributions for *D. melanogaster* it is clear that there is a large overlap of the two score populations and hence the choice of *S*^∗^ necessarily depends on the desired specificity and/or sensitivity. In the simulated data (Figure 4 and Figure 5) we see that haarz generally achieve a high recall (sensitivity), and at the same time offers a high positive predictive value, (PPV) i.e. low false discovery rate. Thus, for the *D. melanogaster* data many sites may be ambiguous to call, and our tool will err on the conservative side to maintain a high PPV.

## Conclusions

We have presented a data adaptive model for variant calling based on easily accessible read characteristics, namely the log-likelihoods of nucleotide qualities, relative read positions, alignment errors, multiple hits and the mismatch rate at a position to obtain a score. With the exception of nucleotide qualities, which are provided as input by the sequencing method, all log-likelihoods are sampled from the data itself. We show that in simulated as well as in real data sets this score gives rise to a mixture distribution that distinguishes between true variants and noise. A spline fit to the overall marginal density allows us to determine a decision threshold that optimally separates these score populations. In the simulated data we demonstrated that this simple model is at par with two of the most commonly used probabilistic models for SNV calling methods in terms of both sensitivity and specificity.

When applying our model to actual next-generation sequencing data, we observe that the distributions of the scores vary significantly among the different data sets. As expected, the clearest separation of the mixture was obtained for the haploid *E. coli* data set. In addition, the small size of the genome and the absence of repetitive elements probably improves the separability of the scores. The situation for the two diploid genomes *A. thaliana* and *D. melanogaster* is different. While both genomes have comparable sizes, the separability of the score distributions varies strongly among these two data sets. While a clear minimum can be found for the plant, the mixture in *D. melanogaster* appears to be more complicated. In this case, by construction our model selects a conservative decision threshold. While the number of calls is similar to the other probabilistic SNV callers in the simulations as well as the next-generations data sets of the plant and the bacteria, it is significantly reduced in the fruit fly data set. These differences indicate that the characteristics of next-generation data sets have a strong impact on the success of variant calling. Furthermore, we observe that, at least for the score proposed here, a significant difference of the separability of the mixture distribution can be found between simulated and real data. Thus, we argue that data adaptive components could help to balance the trade-off between sensitivity and specificity.

## Appendix

### Read simulation

For the simulation of reads and allele contents we used the program GemSIM (v. 1.6). We simulated reads for the human chromosome 21 (hg19) with different coverages using an Illumina specific error model (ill100v5_s).



### Benchmarks and command line parameters

For the benchmarks we have aligned the simulated as well as the real reads with bwa and called the variants with SAMtools and GATK. For our own model the reads were aligned using segemehl. The command line parameters and version numbers are given below. a) BWA v 0.6.2



b) GATK v 2.8.1 (GenomeAnalysisTK-2.8-1-g932cd3a) calling:



filtering:



c) SAMtools v 0.1.19calling:



filtering:



d) segemehl v 0.1.7



obtaining site characteristics (written to sorted.haarz.idx):



obtaining site characteristics for low variant allel frequencies:



calling:


